# Early medieval genetic data from Ural region evaluated in the light of archaeological evidence of ancient Hungarians

**DOI:** 10.1038/s41598-020-75910-z

**Published:** 2020-11-05

**Authors:** Veronika Csáky, Dániel Gerber, Bea Szeifert, Balázs Egyed, Balázs Stégmár, Sergei Gennad’evich Botalov, Ivan Valer’evich Grudochko, Natalia Petrovna Matveeva, Alexander Sergejevich Zelenkov, Anastasiia Viktorovna Sleptsova, Rimma Dmitrievna Goldina, Andrey Vasilevich Danich, Balázs Gusztáv Mende, Attila Türk, Anna Szécsényi-Nagy

**Affiliations:** 1 Laboratory of Archaeogenetics in the Institute of Archaeology, Research Centre for the Humanities, Budapest, Hungary; 2 Department of Genetics, ELTE – Eötvös Loránd University, Budapest, Hungary; 3 South Ural State University, Chelyabinsk, Russia; 4 University of Tyumen, Tyumen, Russia; 5 Tyumen Scientific Centre SB RAS, Institute of the Problems of Northern Development, Tyumen, Russia; 6 Department of History, Archaeology and Ethnology of Udmurtia of the Institute of History and Sociology, Udmurt State University, Izhevsk, Russia; 7 Perm State Humanitarian-Pedagogical University, Perm, Russia; 8 Faculty of Humanities and Social Sciences, Institute of Archaeology, Pázmány Péter Catholic University, Budapest, Hungary

**Keywords:** Archaeology, Phylogenetics, Population genetics

## Abstract

The ancient Hungarians originated from the Ural region of Russia, and migrated through the Middle-Volga region and the Eastern European steppe into the Carpathian Basin during the ninth century AD. Their Homeland was probably in the southern Trans-Ural region, where the Kushnarenkovo culture was disseminated. In the Cis-Ural region Lomovatovo and Nevolino cultures are archaeologically related to ancient Hungarians. In this study we describe maternal and paternal lineages of 36 individuals from these regions and nine Hungarian Conquest period individuals from today’s Hungary, as well as shallow shotgun genome data from the Trans-Uralic Uyelgi cemetery. We point out the genetic continuity between the three chronological horizons of Uyelgi cemetery, which was a burial place of a rather endogamous population. Using phylogenetic and population genetic analyses we demonstrate the genetic connection between Trans-, Cis-Ural and the Carpathian Basin on various levels. The analyses of this new Uralic dataset fill a gap of population genetic research of Eurasia, and reshape the conclusions previously drawn from tenth to eleventh century ancient mitogenomes and Y-chromosomes from Hungary.

## Introduction

The Ural region was involved in numerous migrations, which events also shaped the history of Europe. The archaeological imprint of these events can be witnessed among others by the early medieval cemeteries of the South-Ural region. Compact cemeteries with few hundred tombs are typical of this territory, which have provided rich archaeological findings first over the past 10–15 years^1–5^. According to archaeological, linguistic and historical arguments, the ethnogenesis of modern Hungarian population can be traced back to the Ural region^1,6,7^.

**Figure 1:**
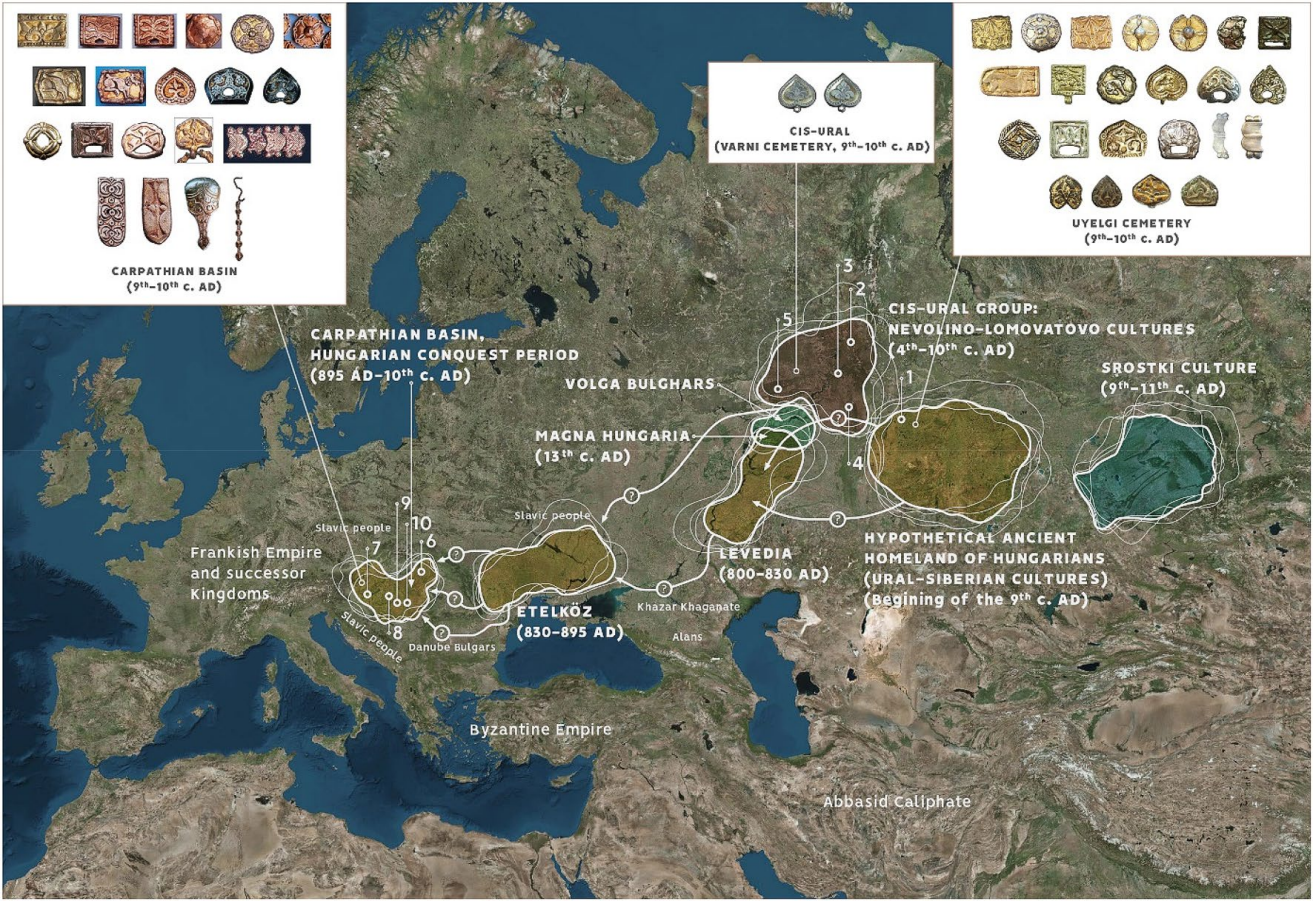
Location of investigated early medieval archaeological sites from South-Ural region and Carpathian Basin, with the possible migration routes, hypothetical Homeland of ancient Hungarians and the similar types of the archaeological finds. Trans-Ural region: Uyelgi cemetery (Kushnarenkovo-Karayakupovo culture) (1); Cis-Ural group: Bayanovo (Late Lomovatovo culture) (2), Brody (Early Nevolino culture) (3), Bartym (Nevolino culture, Phase II) (4), Sukhoy Log (Late Nevolino culture) (5); Hungarian conquerors in the Carpathian Basin: Nyíregyháza-Oross Megapark and the M3 161. burial site (6), Balatonújlak-Erdő-dűlő (7), Harta-Freifelt (8), Kiszombor-Tanyahalom (9), Makó-Igási járandó (10). Similar type of the archaeological finds in the heritage of the Hungarian Conquest period in the Carpathian Basin (ninth–tenth centuries AD) from different sites, in the material of Cis-Ural region (Varni cemetery ninth–tenth centuries AD) and Uyelgi and Sineglazovo cemeteries in Trans-Ural region (ninth–tenth centuries AD). The map of Europe is owned by the Institute of Archaeology in the Research Centre of the Humanities, Budapest, Hungary, and was modified in Adobe 846 Illustrator CS6. The photos were taken by Sergei G. Botalov and Attila Türk.

Based on linguistic evidences, the Hungarian language, belonging to the Ugric branch of the Uralic language family, was developed at the eastern side of Ural Mountains between 1000 and 500 BC^8,9^. According to the written and linguistic sources and archaeological arguments, after the sixth century AD, part of the predecessors of Hungarians moved to the Western Urals (Cis-Ural region) from their ancient homeland. Around the first third of ninth century AD a part of this Cis-Uralic population crossed the Volga River and settled near to the Khazarian Khaganate in the Dnieper-Dniester region^1–5,10^ (Fig. 1). Early Hungarians lived in Eastern Europe (forming the so-called Subbotsy archaeological horizon) until the conquest of the Carpathian Basin that took place in 895 AD. The material traits of tenth century AD Carpathian Basin was rapidly transformed after the conquest, its maintained cultural connections with East-European regions have numerous doubtless archaeological evidence^2,4,11^.

Genetic history of prehistoric to medieval populations of the Ural region have been scarcely investigated to date. On the other side, the populations of the medieval Carpathian Basin have been intensively studied from the perspective of uniparental markers^12,13^. Recently, Neparáczki et al. have published 102 whole mitogenomes from early Conquest period cemeteries in Hungary^14^. Authors have suggested that the mixed population of steppe nomads (Central Asian Scythians) and descendants of the East European Srubnaya culture’s population—among other undescribed populations—could have been the basis of genetic makeup of Hungarian conquerors. Their results furthermore assume Asian Hunnic-Hungarian conqueror genetic connections^14^. It is important to note, that the investigated medieval sample set does not represent the conqueror population as a whole, hence 76% of the samples originated from a special site complex Karos-Eperjesszög from northeast Hungary, which is one of the most important sites of the Hungarian Conquest period with many findings of eastern characteristics as well. The conclusions are large-scale, but the most highlighted connection with the population of the Srubnaya culture is vague, because it existed more than 2000 years before the appearance of the first traces of ancient Hungarians’ archaeological heritage. Additionally, further mentioned relations such as the Xiongnu (Hunnic) genetic dataset is bare from Eurasia, and Huns’ genetic heritage is yet uncharacterised.

Two recent articles have investigated the Y-haplogroup variability of Hungarian conquerors describing the conqueror’s elite population as heterogenous, with significant proportion of European, Finno-Permic, Caucasian and Siberian (or East Eurasian) paternal lineages^15,16^. Fóthi et al. have claimed that the Hungarian conquerors originated from three distant sources: Inner Asia (Lake Baikal—Altai Mountains), Western Siberia—Southern Urals (Finno-Ugric peoples) and the Black Sea—Northern Caucasus (Northern Caucasian Turks, Alans, and Eastern Europeans)^15^. Both studies^15,16^ pointed out the presence of the Y-haplogroup N-Z1936 (also known as N3a4-Z1936 under N-Tat/M46), which is frequent among Finno-Ugric speaking peoples^17^. This lineage also occurs among modern Hungarians in a frequency up to 4%. Post et al. have reconstructed the detailed phylogeny of N-Z1936 Y-haplogroup showing that specific sublineages are shared by certain ethnic groups, e.g. N-Y24365/B545 by Tatars, Bashkirs and Hungarians, which connect modern-day Hungarians to the people living in the Volga-Ural region^17^.

Earlier mitochondrial DNA (mtDNA) studies of modern populations speaking Uralic languages suggest that the distribution of Eastern and Western Eurasian mtDNA lineages are determined by geographic distances rather than linguistic barriers^18–20^, e.g. Finno-Ugric populations from Volga-Ural region seem to be more similar to their Turkic neighbours than to linguistically related Balto-Finnish ethnic groups^18^. The recent study of 15 Uralic-speaking populations describes their similarities to neighbouring populations as well, however they also share genetic component of possibly Siberian origin^21^. Even though that some mitochondrial lineages of present-day Hungarians have possible Siberian descent^22^, the Hungarians’ gene pool differs from that of other Uralic-speakers^21^.

The main goal of this study is to expand the current set of archaeological knowledge about the early medieval populations of the Ural region by archaeogenetic methods. During the collection of 36 human samples from Ural region processed in this study, the most important intention was to collect burials exclusively from such professionally excavated and appropriately documented cemeteries from the South-Ural region, which are culturally and temporally (directly or indirectly) connected to the ancestors of Hungarians (Fig. 1 and Supplementary Fig. S1a-h).

The sampled Uyelgi cemetery from Trans-Ural region presented the greatest similarity to the archaeological traits of the tenth-century Carpathian Basin (Fig. 1, Supplementary Fig. S1e-h). This cemetery of the late Kushnarenkovo culture was used from the end of eighth century to the eleventh century^2,23^.

As the archaeological and historical theories are slightly diverse, we aimed to cover a wide range of early medieval archaeological cultures located in the middle course of the Kama River in the west side of the Ural Mountains (Cis-Ural region). Scholars connect the termination of the Nevolino Culture in eighth–ninth centuries AD to the westward migration of ancestors of Hungarians^1–3^, hence the sampling was carried out in all three phases of this culture: Brody (third–fourth centuries), Bartym (fifth–sixth centuries) and Sukhoy Log (seventh–eighth centuries)^24^ (Fig. 1). Furthermore, we investigated the Bayanovo cemetery (ninth–tenth centuries AD), which represents the southern variant of Lomovatovo culture^3^ that shows close cultural connection to its southern neighbour Nevolino culture. The sampling of the richly furnished graves of Bayanovo was limited by the poor preservation of bone samples (see Supplementary text, Figs S1b-d)^6^.

Additionally, we reanalysed nine samples from tenth- to twelfth-centuries ancient Hungarians for whole mitogenomes from the Carpathian Basin, who were chosen from the previous study Csősz et al.^13^ based on identical hypervariable I region (HVRI) haplotypes of mtDNA with some of investigated Uralic individuals.

In this paper, our main purpose was to characterize the maternal and paternal genetic composition of populations from the third- to eleventh-centuries South-Ural region and compare the results with the available ancient and modern genetic datasets of Eurasia. We also aimed to describe possible genetic connections between the studied Uralic populations and the Conquest period populations of the Carpathian Basin.

## Results and discussion

The sample-pool consisted of 29 males and 16 females. We performed whole mitochondrial DNA and 3122 nuclear SNPs target-enrichment combining with shallow shotgun sequencing. With the latter we obtained autosomal and Y-chromosomal SNPs, as well as sex-determination of 45 individuals that originated from five different cemeteries in Ural region and six burial sites in present-day Hungary (Carpathian Basin). Furthermore, we investigated the Y-STR profiles of 20 male individuals from the Ural-region. For detailed information see Supplementary Tables S1, S2, S13. For the radiocarbon dating and stable isotope data see the Supplementary Information chapter 2 and Supplementary Tables S1, S14.

### Primary observations

**Figure 2:**
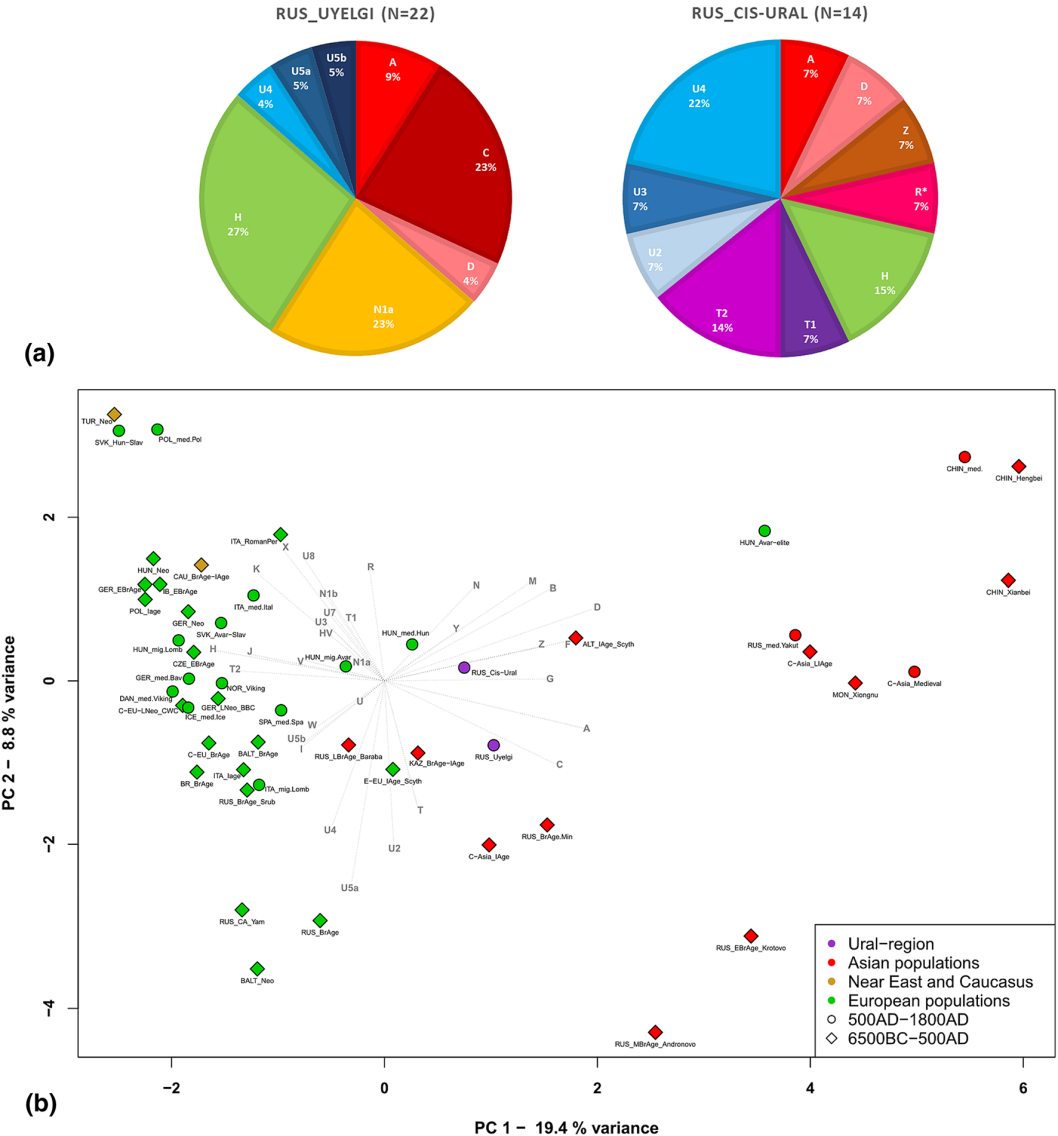
Mitochondrial haplogroup frequencies of the investigated populations from Ural region (**a**) and PCA plot with 50 ancient populations, representing first and second principal components (**b**). (**a**) The characterized populations: cemetery Uyelgi (RUS_Uyelgi) from the Trans-Ural region (see Supplementary Tables S2 and S3); cemeteries Bayanovo, Sukhoy Log, Bartym and Brody (Nevolino-Lomovatovo cultures) grouped into “Cis-Ural” (RUS_Cis-Ural). (**b**) PCA analysis based on haplogroup frequencies in Eurasian ancient populations. Clear separation of Asian (red) and European (green) populations is visible on the plot, the investigated Cis-Ural and Uyelgi sites (violet-coloured) are located between them: the Cis-Ural (RUS_Cis-Ural) near to the Hungarian conquerors (HUN_med.Hun) and the Uyelgi (RUS_Uyelgi) is positioned between the Iron Age population from Central Asian Steppe (C-Asia_IAge), Russian Bronze Age population from Minusinsk Depression (RUS_BrAge.Min), Bronze Age and Iron Age populations from Kazakhstan (KAZ_BrAge-IAge) and the East European Iron Age Scythians (E-EU_IAge_Scyth).

45 high coverage mitochondrial genomes were obtained (sequencing depth from 8.71 × to 154.03 ×), with mean coverage of 71.16 × and an average contamination rate of 0.2%. The new dataset consists of the mixture of nine macrohaplogroups (A, C, D, H, T, U, N, R, Z) (Fig. 2a). Haplogroups of presumably West Eurasian origin are represented by U (U2e1, U3a1, U4a1d, U4b1a1a1, U4d2, U5a1a1, U5b2a1a1, N = 12), H (H1b2, H3b, H40b, N = 9), N (N1a1a1a1a, N = 5) and T (T1a1, T1a2, T2b4h, N = 5), although phylogeographic analyses show eastern origin for some of them, see Supplementary Table S16 and Supplementary Fig. S4a-s. Eastern Eurasian lineages are represented by A (A + 152 + 16,362, A12a, N = 4), C (C4a1a6, C4a2a1, N = 6), D (D4j, D4j2, N = 2), along with R11b1b and Z1a1a by one individual each (Fig. 2a).

Even though that the Hungarian conquerors were selected based on mtDNA HVRI matches with certain ancient individuals from the Ural region, they have not proved to be identical on whole mitogenome level, but remained phylogenetically close to the associated samples (see Supplementary Fig. S4a-s).

A few mitochondrial lineage relations connect Trans-Ural and Cis-Ural regions: e.g. samples from Uyelgi and Sukhoy Log clustered together in one main branch of the A + 152 + 16,362 haplogroup tree (Supplementary Fig. S4b), furthermore samples from Uyelgi and Brody (with haplogroup D4j2) and from Uyelgi and Bartym (with haplogroup U4d2) are located on the same main branch as well (Supplementary Figs. S4e and S4p).

In contrast to the mitochondrial lineages, the Y-chromosomal gene pool based on STR and/or SNP data show homogenous composition in our dataset: 83.3% is N-M46, 5.5% G2a (G-L1266), 5.5% J2 and 5.5% is R1b of the typed male individuals (Supplementary Table S2). 13 male samples out of 19 from Uyelgi cemetery carry Y-haplogroup N with various DNA preservation-dependent subhaplogroup classifications, while in the Cis-Ural we detected three N-M46 Y-haplogroups (samples from Brody, Bartym and Bayanovo cemeteries). The overall poor preservation of further Cis-Uralic samples from Sukhoy Log and Bartym disabled further Y-chromosome-based analyses (Supplementary Table S2).

### Comparative population genetic analyses of maternal lineages and genomic data

We performed population genetic statistical analyses as well. The principal component analysis (PCA) and Ward clustering of 50 ancient and 64 modern populations were performed separately (Fig. 2b, Supplementary Figs. S5–S8), based on haplogroup frequencies (Supplementary Tables S3, S4). The Hungarian conquerors are the closest population to the Cis-Ural group on the PCA (along PC1 and PC2 components, see Fig. 2b) and this population is relatively near to the Uyelgi among the Iron Age population from Central-Asia and the East European Scythians along PC1 and PC3 components (Supplementary Fig. S5), because these ancient populations have mixed pool of Western and Eastern Eurasian macrohaplogroups, which is unusual in European and Asian populations that are separated along the PC1. The nearby position of Cis-Ural and Uyelgi to the Hungarian conquerors is displayed on the mtDNA haplogroup-based Ward type clustering tree too, where they appear in the same main branch (Supplementary Fig. S6). Some of Central-South Asian and Finno-Ugric modern populations (e.g. Khanty and Mansi) show close connections to the investigated Cis-Ural and Uyelgi populations based on Ward-type clustering and PCA (Supplementary Figs. S7 and S8). Pairwise F_ST_ values based on whole mitogenome sequences indicate non-significant differences of the Cis-Ural from 13 ancient populations (Supplementary Table S5), among them the Hungarian conquerors^14^ show the lowest genetic distance (F_ST_ = 0.00224) (for further F_ST_ values, *p* values, and references see Supplementary Table S5).

The genetic distances of Hungarian conquerors and the investigated two populations from the Ural region does not correlate with their geographic distances (see Mantel test in Supplementary Table S12). The genetic distance between Uyelgi and Hungarian conquerors is lower than between Uyelgi and geographical closer Cis-Ural population (Supplementary Table S5).

According to the MDS plot of 28 ancient populations based on linearized Slatkin F_ST_ (Supplementary Fig. S9a), the Cis-Ural population shows affinities among others to the populations of medieval Hungarian conquerors along coordinates 1 and 2, and is situated between European and Asian populations, which reflects the raw F_ST_ values. The Uyelgi is standing on the Asian part of the plot relatively far from all ancient populations, which is most likely due to its significant and larger genetic distances from ancient populations (except the Late Iron Age population from Central Asia^25^) and the scarcity of Asian comparative mitogenome datasets. The rank correlation heatmap (Supplementary Fig. S9b) of the F_ST_ values of ancient populations supports the MDS plot, where the Uyelgi and Cis-Ural populations cluster with the same ancient populations that are close to them on the MDS plot. The genetic connection of Cis-Ural population and Hungarian conquerors^14^ is obvious based on pairwise F_ST_ calculation and is visible on the PCA and MDS plots as well, where they are the closest, although direct phylogenetic connections are scarce. This indicates geographical proximity of their former settlement area, rather than a direct connection. Neparáczki et al. have described the Hungarian conqueror mitogenome diversity in essence as a mixture of Srubnaya culture associated and Asian nomadic populations. Their analyses and interpretation were restricted by the lack of ancient samples from the Ural region, whereas new data now refine such previous conclusions^14^. Furthermore, it is notable, that the previously studied Hungarian conqueror population is a pool of mixed origin including not only immigrants but also local admixed lineages from the Carpathian Basin.

The Cis-Ural population reveals non-significant genetic distances from four modern populations of Central Asian Highlands, furthermore seven populations of Near East and Caucasus region and six European populations (see Supplementary Table S6) indicating a mixed character of this population, which is also visible on the MDS plot (Supplementary Fig. S10).

Interestingly, the mitogenome pool of Uyelgi shows significant differences in genetic distances among nearly all prehistoric and modern populations including Hungarian conqueror population in spite of the extensive phylogenetic connections, which might be explained by high amount of related lineages within the population, as well as by their mixed character of Eastern- and Western-Eurasian haplogroups.

**Figure 3:**
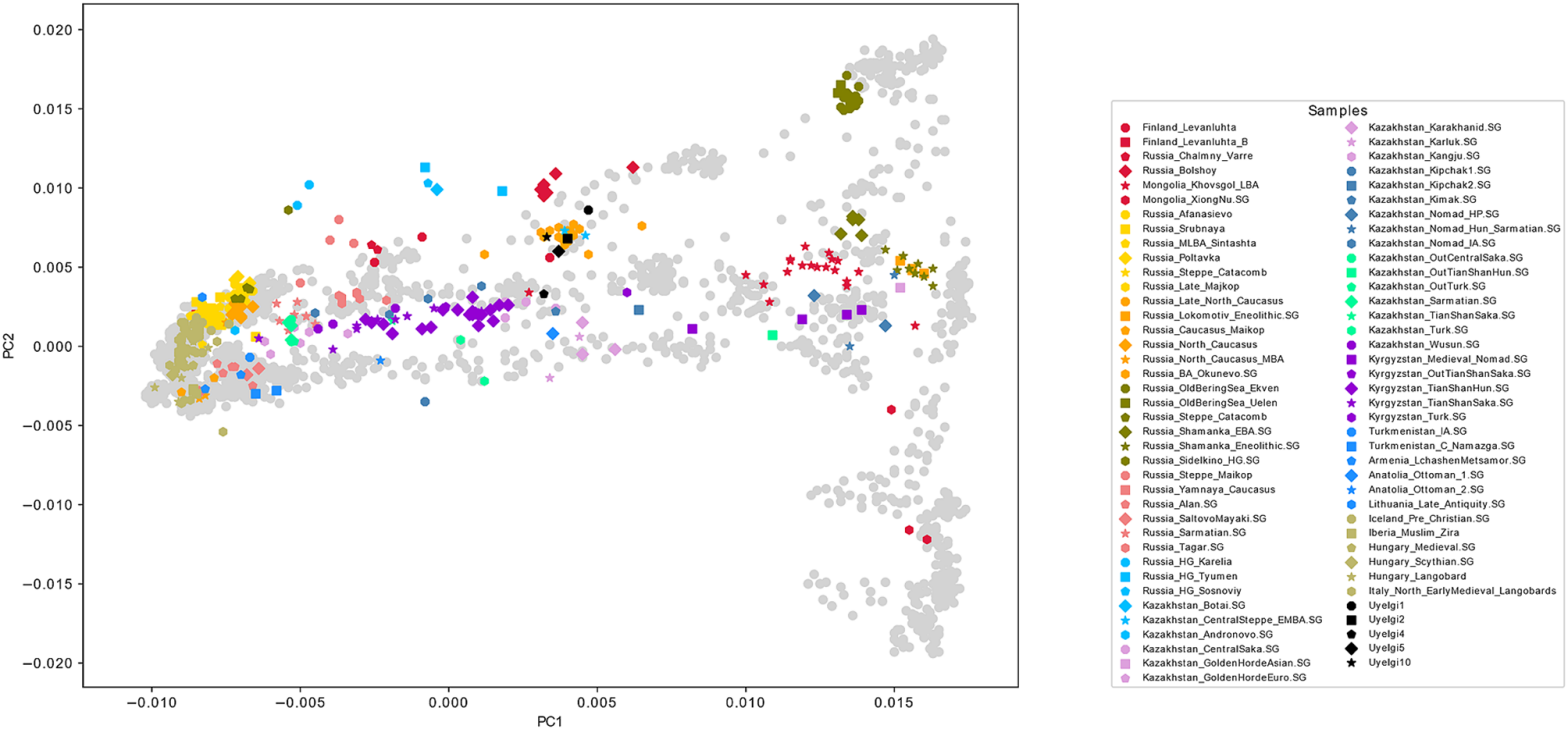
Genomic PCA with projection of five ancient genotypes from Uyelgi site and other ancient genotypes to the modern individuals along PC1 and PC2. PCs were computed by smartpca, on PC1 5.08% and on PC2 0.61% of variance is explained. The Uyelgi samples are plotted between European and Asian populations in the middle, rather in the Uralic stream, close to the modern Bashkirs, Siberian Tatars, Mansis, Aleuts, Selkups (see also Fig. S3a-e), as well as to the ancient populations: Altaian Bronze Age Okunevo population, Bronze Age population from Kazakhstan Central Steppe and Middle Bronze Age Bolshoy population from Russia.

We performed genomic PCA with five Uyelgi samples yielding 10,928 nuclear genomic SNPs on average gained from 3000 SNP capture and shallow shotgun sequencing data (called from 598,094 SNPs of which 524,301 were used for the calculations, see also Supplementary Information, Chapter 3). The PCA plots (Fig. 3, Supplementary Fig. S3a-e) reflect the geographical location of modern populations. The PC1 separates Western and Eastern Eurasians, where Central Eurasians are located in the middle. The PC2 separates Europeans from Southwest Asians, as well as the Eastern Eurasians along the north–south cline. The five Uyelgi samples are plotted together on the genomic PCA (Fig. 3) between European and Asian populations in the centre. They appear along PC1–PC2 rather in the Uralic stream, close to the modern Mansis and Selkups but also to Bashkirs, Siberian Tatars (from the northern groups of steppe-forest, Zabolotniye), and Aleuts. Along PC3 Siberian Tatars are the closest group to the Uyelgi cluster (see Supplementary Fig. S3d). If other ancient populations are projected on the PCA plot, the Uyelgi samples cluster with the Altaian Bronze Age Okunevo population^26^, as well as with the Bronze Age population from Kazakhstan Central Steppe^26^ and are also near to the Middle Bronze Age Bolshoy population from Russia^27^ along first two components (Fig. 3). Based on linguistic clines described by Jeong et al.^28^, the Uyelgi population is plotted between the Uralic speaking individuals and the Turkic language speaking populations from Central Steppe.

Since PCA may not reveal population stratification we performed unsupervised ADMIXTURE (K = 16) on an enlarged sets of SNPs (SI, chapter 3). The five Uyelgi samples with an average calling of 22,540 SNPs show the most similar ancestry cluster proportions to present-day Mansis and Irtysh-Barabinsk Tatars and to a set of various ancient genomes from the Central Steppe region^26^.

To disentangle the connections between these populations and possible population genetic events of thousands of years between populations under study, more ancient reference samples and deeper sequencing is needed.

### The genetic continuity between the horizons of the Uyelgi cemetery (Trans-Ural region)

**Figure 4:**
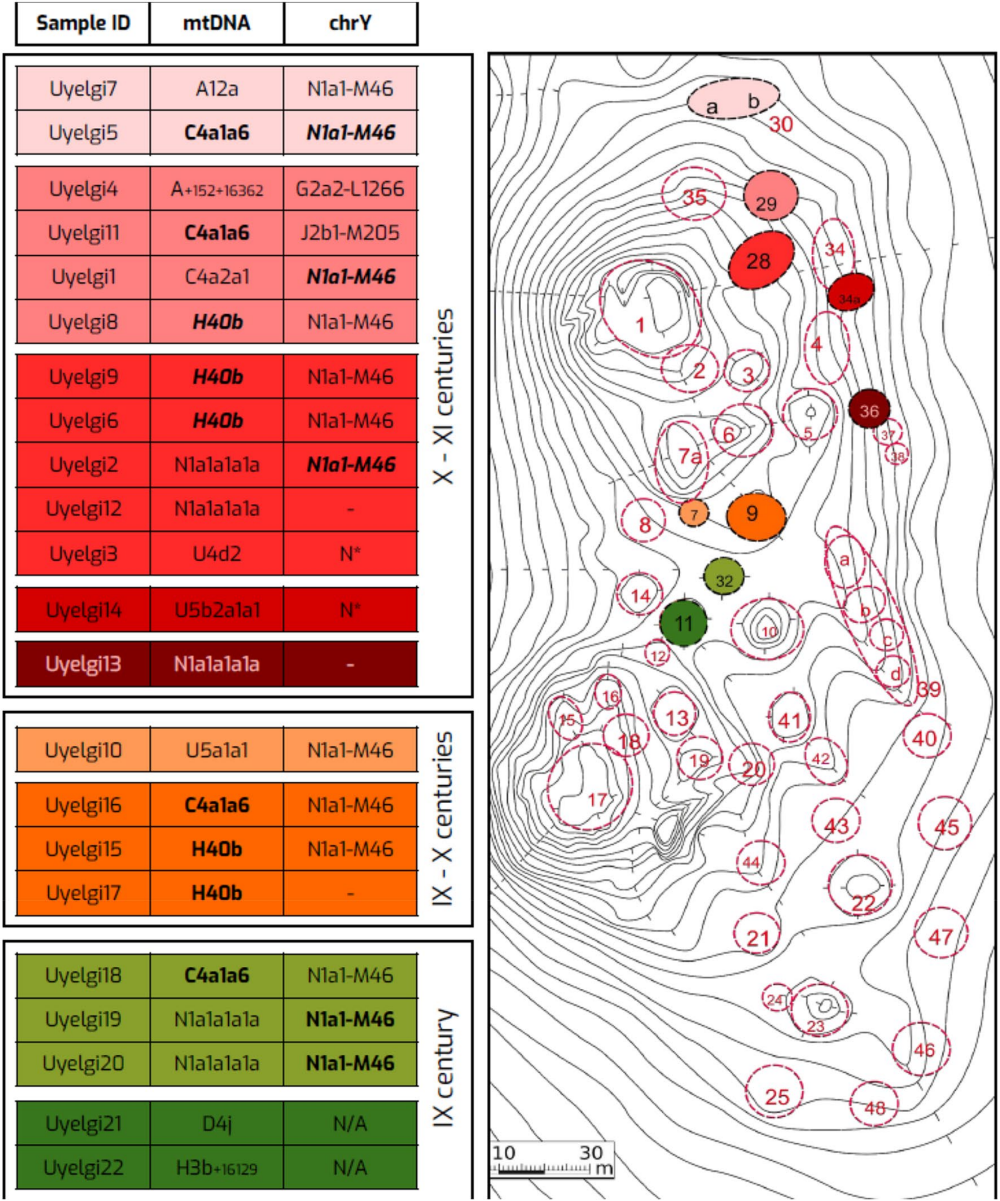
Mitochondrial haplotype and Y-chromosomal similarities between the kurgans of the three horizons of Uyelgi cemetery. Three chronological horizons were defined in the cemetery: an oldest horizon from ninth century (marked with green), a middle horizon from ninth–tenth centuries (marked with orange) and the youngest horizon from tenth–eleventh centuries (marked with red colour). The bold and italic highlighted letters indicate different mitochondrial and/or Y-STR haplotype matches within and between the kurgans whose localization is visible on the right part of the figure. Four identical mitogenome haplotypes belonging to C4a1a6 haplogroup appear in all three horizons in four different kurgans, furthermore two identical haplotypes of H40b haplogroup are from the middle horizon and three also identical but different from the previous haplotypes of H40b come from two kurgans of the youngest horizon. Additionally, the five individuals with mtDNA haplogroup N1a1a1a1a are distributed in three kurgans from the oldest and youngest horizons.

**Figure 5:**
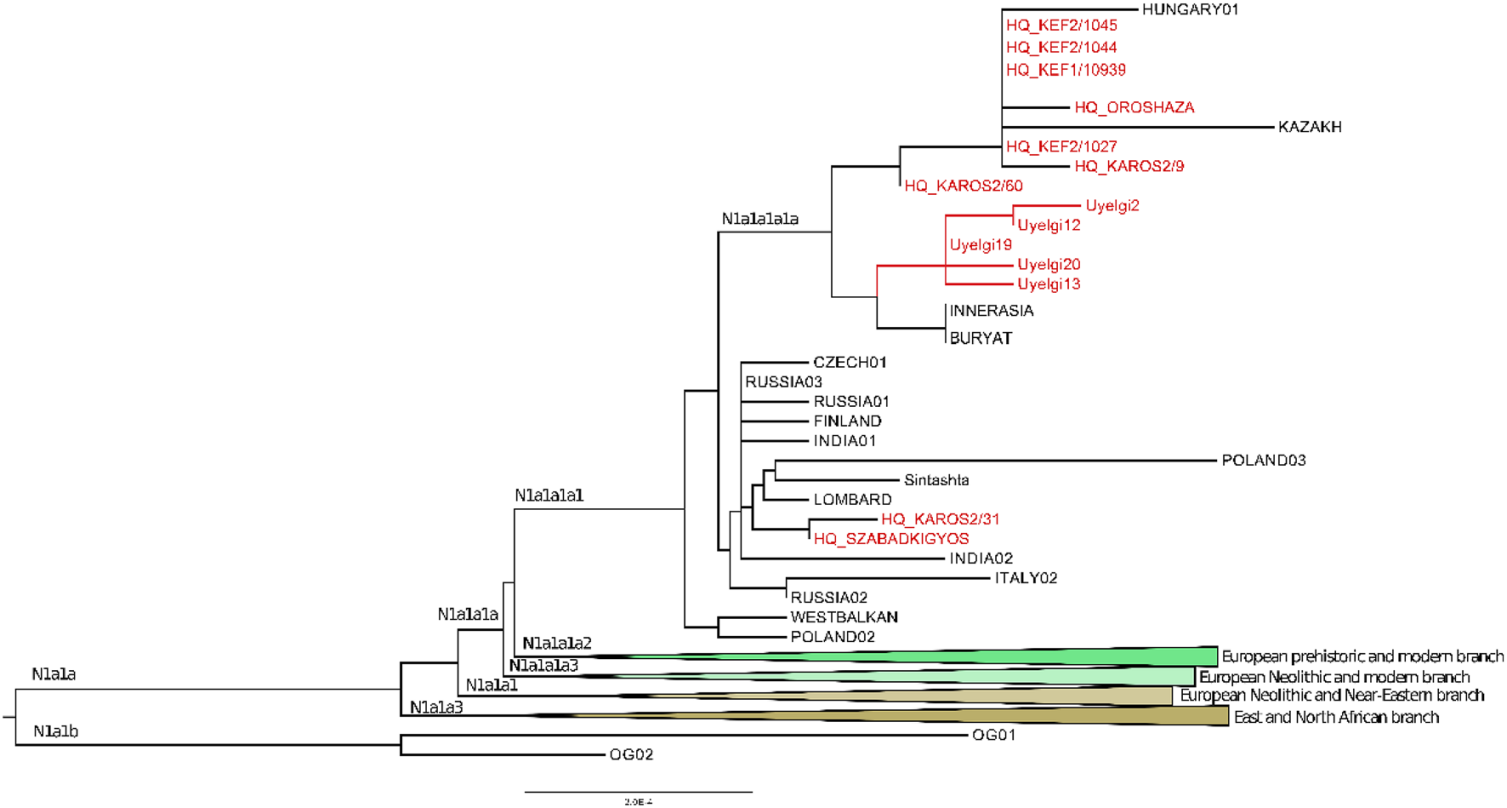
Phylogenetic tree of mitochondrial haplogroup N1a1. The subhaplogroup N1a1a1a1a was detected in five individuals assigned to two horizons of Uyelgi cemetery: Uyelgi19 and Uyelgi20 from the oldest (ninth century) horizon and the Uyelgi2, Uyelgi12 and Uyelgi13 from the youngest horizon (tenth–eleventh centuries), furthermore, in nine tenth centuries Hungarian conqueror graves from various cemeteries in Hungary, and in one modern Hungarian individual. The Uyelgi branch of the tree is very compact, clearly connects the oldest and youngest horizons together, however, the maternal lineages of the populations from Uyelgi and the Hungarian conquerors are separated (for the abbreviation and further information see Table S7).

**Figure 6:**
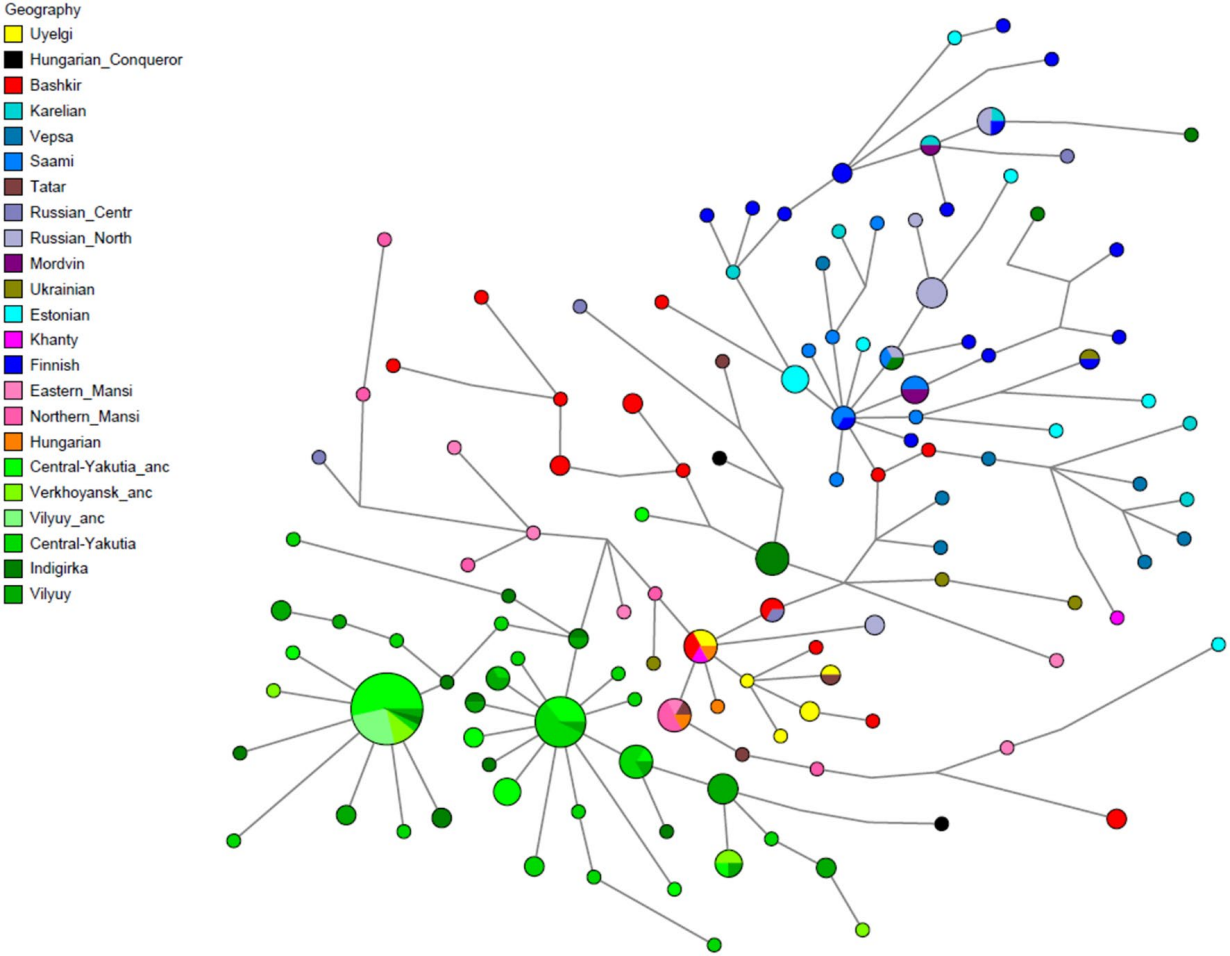
Median Joining network analysis of the N-M46 Y-chromosomal haplogroup based on 17 STRs in 238 samples. All seven samples from Uyelgi (marked with yellow) besides two-two identical samples (Uyelgi1–Uyelgi5 and Uyelgi19–Uyelgi20) are one-step neighbours of each other, as well as to five Mansi, four Bashkir, two Hungarian samples, one Tatar sample from Volga-Ural region and one Central Russian sample. The Uyelgi1 and Uyelgi5 share identical STR haplotype with two present-day Bashkir individuals from Volga-Ural region, one Khanty individual from Western-Siberia and one Hungarian individual. Uyelgi16 has identical STRs with a Tatar individual from Volga-Ural region (see Supplementary Table S8).

The kurgan burials at Uyelgi site can be divided into at least three chronological horizons: (1) the oldest ninth-century, (2) ninth- and tenth-centuries and (3) tenth- and eleventh-centuries according to the archaeological records (see Supplementary text chapter 1 and Supplementary Fig. S1e-h). Uniparental genetic markers show genetic continuity between these horizons suggesting maternally rather endogamous population, which could not be observed in archaeological findings due to high number of disturbed burials in the cemetery. Mitochondrial phylogenies of N1a1a1a1a, C4a1a6 and H40b provide identical or monophyletic lineages within and between the three horizons (see Figs. 4, 5 and Supplementary Fig. S4c, g–h), which trend is more pronounced by haplotype and network analysis of paternal lineages (Fig. 6, Supplementary Figs. S11–S12).

The haplotypes of N-M46 Y-haplogroup are presented in all three horizons, however with little differences in STR profiles (Supplementary Table S2). The oldest and the middle horizons contain only N-M46 haplotypes including two identical STR profiles in Kurgan 32 (ninth century). Three identical Y-STR profiles are detected among individuals of Kurgans 28, 29 and 30 (Figs. 4, 6). Probably further identical Y-haplotypes could have been in this cemetery, but the preservation does not let us reconstruct whole Y-STR profiles of seven males (see Supplementary Table S2). Based on these results we suggest that Uyelgi cemetery was used by a patrilocal community.

Uniparental markers as well the grouping of the graves suggest blood relationships between the investigated individuals at least to some extent. Unfortunately, informative positions for kinship analysis do not exceed 400 autosomal SNPs on pairwise comparisons, thus only conjectures can be made at this level.

### The possible maternal genetic connection of South-Ural region’s populations and the Hungarian conquerors

The genetic connection of Uyelgi cemetery in the Trans-Ural and tenth century Hungarian conquerors in the Carpathian Basin is supposed by close maternal relationships of the following individuals: Uyelgi3 and three Hungarian conquerors from Karos II cemetery^14^ have identical U4d2 mitogenome haplotype (Supplementary Fig. S4p). Furthermore, the mtDNA A12a lineage of Hconq3 (30–40 years old woman from Harta cemetery dated to the first half of tenth century AD) is an ancestor of the mtDNA lineage of Uyelgi7 (see Supplementary Fig. S4a).

The mentioned graves from Uyelgi show the archaeological characteristic of the Srostki culture, where the gilt silver mounts with plant ornaments were typical, and which was disseminated from the Siberian Minusinsk Depression and the Altai region through the Baraba Steppe and North-Kazakhstan to the Trans-Ural region (Fig. 1). Moreover, it is notable that the archaeological findings in these kurgans are dated not earlier then the tenth century AD, i.e. after the Hungarian conquest of the Carpathian Basin. Identical mitogenome sequences with the Hungarian conquerors from Karos cemetery appearing on the phylogenetic tree U4d2 can point out close biological connections or common source population of the Uyelgi population and the Hungarian conquerors.

The D4j phylogenetic tree shows one interesting phenomenon: Uyelgi21 clusters with one modern-day Hungarian (Fig. S4e). The findings of this Kurgan 11 (belonging to the Srostki culture) show similarities to the typical findings of the Hungarian conquerors from the Carpathian Basin as well (see Fig. 1 and Supplementary Fig. S1h).

The mitogenome of individual Uyelgi10 and three identical lineages of two Hungarian conquerors (Hconq1 and Hconq6) from Balatonújlak-Erdő-dűlő and Hconq9 from Makó-Igási járandó cemetery clustered together in one branch on the phylogenetic tree of haplogroup U5a1a1 (Supplementary Fig. S4q). The Uyelgi10 shows mixed character from archaeological point of view: the findings can be connected to the ninth century AD as well as to the cultural influences of the Srostki culture (for the detailed information see Supplementary information)^29,30^. The samples of adult women from Balatonújlak-Erdő-dűlő buried with gilt silver hairpins could be dated (based on archaeological findings) to the middle third of the tenth century AD^31^. One of the burials had a grave with a sidewall niche of eastern origin. The grave from Makó-Igási járandó without findings is dated to the middle third of eleventh century AD, i.e. to the Árpádian Age, when conquerors and the local population presumably admixed already. Interestingly, the 25–30 years old man shows some Asian cranial traits as the most men buried in this cemetery^32^.

The connection of Uyelgi cemetery and Hungarian conquerors is visible on the N1a1a1a1a branch of the tree of mtDNA haplogroup N1a too, that was prevalent among the ancient Hungarians (Fig. 5). Here seven Hungarian conqueror samples from cemeteries Kenézlő-Fazekaszug, Orosháza-Görbicstanya and Karos-Eperjesszög clustered on one branch, while the five Uyelgi samples from the earliest and latest horizons are located on the adjacent branch. These results signalize indirect connection between these two populations and their common source in agreement with the archaeological chronology of Uyelgi site.

The maternal genetic connection of the Cis-Ural region and the Hungarian conquerors is apparent especially on the phylogenetic tree of mitochondrial haplogroup T2b4h, where Bartym2, Bay3 and Hungarian conqueror from Karos site^14^ are located on the same branch, moreover, the individuals from Bartym and Karos share the same lineage that is ancestral to the mtDNA lineages of individual from Bayanovo (Supplementary Fig. S4k). The lineage of Karos sample was determined as of possibly Asian origin^14^, nevertheless, this assumption is revisited by our data, not only by actual phylogenetic connections but due to the recurrent western presence of these lineages even from pre-medieval times.

The described phylogenetic connections of six mitochondrial lineages between Ural region and Hungarian conquerors signal rather close maternal genetic connection between these populations, which is supported by the archaeological findings, as well.

### Ancient paternal lineages of the South-Ural region

Majority of Uyelgi males belong to Y chromosome haplogroup N, and according to combined STR, SNP and network analyses they belong to the same subclade within N-M46 (also known as N-Tat and N1a1-M46 in ISOGG 14.255). N-M46 is geographically widely distributed from East of Siberia to Scandinavia^33^. One of its subclades is N-Z1936 (also known as N3a4 and N1a1a1a1a2 in ISOGG 14.255), which is prominent among Uralic speaking populations, probably originated from the Ural region as well and mainly distributed from the west of Ural Mountains to Scandinavia (Finland). Seven samples of Uyelgi site most probably belong to N-Y24365 (also known as N-B545 and N1a1a1a1a2a1c2 in ISOGG 14.255) under N-Z1936, a specific subclade that can be found almost exclusively in todays’ Tatarstan, Bashkortostan and Hungary^17^ (ISOGG, Yfull). Median Joining (MJ) network analysis is performed using 238 N-M46 Y-haplotypes including seven samples from Uyelgi detected with 17 STR loci (Fig. 6, Supplementary Table S8) as well as 335 N-M46 Y-haplotypes with 12 STR loci (Supplementary Fig. S12, Supplementary Table S8). Based on MJ of 17 Y-STR loci, certain samples show identical or one-step neighbour profiles to Bashkirs, Khantys^17^, Hungarians^34^, Tatars from Volga-Ural region and a Central Russian sample^17^ (Fig. 6). The MJ based on 12 Y-STR data shows one-step neighbour connection of Uyelgi with two Hungarian conquerors from Bodrogszerdahely-Bálványhegy and Karos-Eperjesszög^15^ (Supplementary Fig. S12). YHRD online database shows further affinities or identities among Finnish, Ural region (Sverdlovsk Oblast) or European Russian region (Penza and Arkhangelsk Oblasts) samples, notably either from territories of Uralic language affinities, where also the Hungarian language belongs to, or along the supposed migration route of early Hungarians.

It is noteworthy that the seventh-century Avar elite males from the Carpathian Basin^35^, in spite of the similar N-M46 frequency to Uyelgi, had a distant subtype (N-F4205, N1a1a1a1a3a in ISOGG 14.255), which is prominent in present-day Mongolic speaking populations around Lake Baikal^33^. Furthermore, they had a fairly different population history than populations of this study, therefore they shall not be confused with each other^35^.

Uyelgi11 belongs to J2 Y-haplogroup. The Y-haplogroup J is widespread nowadays and probably descended from the Near East^36^. Interestingly, a Hungarian conqueror from Sárrétudvari-Hízóföld (SH/81) carries the J2a1a subgroup^16^, however Uyelgi11 could not be typed downstream to J2 and therefore further assumptions cannot be made at this level.

Uyelgi4 belongs to G-L1266 (G2a2b2a1a1a1b in ISOGG 14.255), which sublineage is confirmed to be present outside of Europe within the European G-L140 branch of G. Among Hungarian conquerors the presence of G-L30 (G2a2b in ISOGG 14.255) was attested by Neparáczki et al.^16^ from Karos II (K2/33) without further classification or STR data, but recently G-L1266 has been confirmed by Fóthi et al.^15^ which sample could also be included in our STR analysis. By using 14 STR markers in this case, due to the limitations of the database, MJ network shows a Caucasian affinity of both Hungarian conqueror (RP/2) and Uyelgi individuals (Supplementary Fig. S11, Supplementary Table S9), however, neither identity nor monophyly can be observed between them.

## Conclusions

The Ural region had an important role in ancient Hungarians’ ethnogenesis based on archaeological, linguistic and historical sources, although the results of these research fields exhibit differences of chronological and cultural aspects. The new mitogenome, Y-chromosome and shallow shotgun autosomal DNA sequence data from the South-Urals presented here confirms the region’s relevance from population genetic perspective too.

The overall maternal makeup of the investigated 36 samples from the Ural region in a phylogenetic and phylogeographic point of view suggests a mixed characteristic of rather western and rather eastern components, although the paternal lineages are more homogenous with Y-haplogroups typical for the Volga-Ural region. The exact assignment of each mitochondrial haplotype of the Trans-Uralic Uyelgi population to the Eastern and Western Eurasian components is impossible, but comprehensive representatives are present. Mitochondrial haplogroups of European origin N1a1a1a1a and H40b provide a horizon-through success of maternal lineages with inner diversification, which suggests a base population of a rather western characteristics. On the other hand, identical (C4a1a6) or single (A, A12a, C4a2a1) haplotypes with strong eastern phylogeography, highly pronounced in the third horizon, suggest a relatively recent admixture to this population. The apparent co-occurrence of genetic and archaeological shift is however contradicted by the homogeneity of ancestry components, nuclear genomic PCA positions, homogeneity of paternal makeup (although this one itself can be explained by patrilocality), and presence of eastern component (C4a1a6) in all horizons. Despite the fact that the genetic contribution of a population related to the Srostki culture cannot be excluded at this level, it is more likely that the majority of eastern components admixed before the usage of the Uyelgi cemetery. The uniparental genetic composition of Uyelgi population signals them as a chronologically and/or geographically related population to the possible genetic source of the Hungarian conquerors. Furthermore, their preliminary autosomal results show that they shared their allele frequency makeup with modern Uralic and West Siberian populations that are linguistically or historically related to Hungarians, which provide a good standpoint for future studies.

The maternal phylogenetic connections of Uyelgi with Hungarian conquerors can be divided to indirect (monophyletic but not successive) and direct (identical or one-step neighbour) relationships. Interestingly, indirect connections can be genetically assigned to the western-characteristic base population, whereas direct connections are almost exclusive to the admixed eastern component. One possible explanation for this phenomenon is that Hungarian conquerors and Uyelgi shared common ancestry in the past that separated prior eastern admixture, latter which provided genetic components subsequently to both groups. The exact origin or identification of the eastern component yet to be described, however, nuclear admixture proportions and loose phylogenetic connections points towards Central Asia.

The phylogenetic makeup of Cis-Ural region questions their compactness or successiveness; however, the scarce data does not allow extensive analysis for this group. Hungarian conqueror lineage-based connections here are sporadic, but regional affinity is observable, which is more pronounced in MDS and PCA. Earlier studies based solely on the genetic makeup of Hungarian conquerors tend to connect the non-European lineages to various eastern regions, but especially the presence of rare Eastern Eurasian haplotypes in the Late Iron Age and Early Medieval Cis-Ural group may reshape these conclusions in the future.

In subsequent studies, we plan to extend the presented dataset with high coverage genomic analyses and including samples from further East European cemeteries of ancient Hungarians and their neighbouring communities.

## Material and methods

### Sampling

Our aim was to collect samples from all available anthropologically well characterised human remains from five cemeteries of the Ural region: from Uyelgi 22 samples, from Bayanovo (Boyanovo) three samples, from Sukhoy Log five samples, from Bartym five samples and from Brody one sample, as well as nine comparative samples from Carpathian Basin (for more information see Supplementary Table S1).

Individuals from the cemeteries Bayanovo, Sukhoy Log, Bartym and Brody were grouped as “Cis-Ural” in the mtDNA population genetic analyses, indicated by the relative geographical proximity (~ 400 km) and archaeological similarities (see Supplementary text). Furthermore, these cemeteries are connected to Hungarian prehistory through various archaeological evidences and historical sources as well^1,3^.

### Sample preparation

All procedures were performed in a dedicated ancient DNA laboratory according cleanness recommendations at Laboratory of Archaeogenetics, Institute of Archaeology, Research Centre for the Humanities in Budapest, Hungary. After photo documentation and bleach, samples were UV-C irritated for 30 min per side. Therefore, samples were abraded by using bench-top sandblaster machine with clean sand, followed by additional UV-C exposure procedure for 20 min per side. Cleaned bone samples were grinded into fine powder. Approximately 100 mg (80–120 mg) of powder was collected and processed^13,37^.

### DNA extraction, library preparation and NGS sequencing

DNA extraction was performed according the protocol of Dabney et al.^38^ with minor changes pointed also by Lipson et al.^37^.

For verifying the result of DNA extraction, a test PCR reaction was performed^37^. DNA library preparation with partial uracil-DNA-glycosylate treatment was performed as described at Rohland et al.^39^ with minor modifications. Partially double-stranded and barcoded P5 and P7 adapters were used. The libraries were amplified using TwistAMP (TwistDX) in 34.3 µL final volume. Amplification reaction products were purified by AMPure Beads Purification (Agilent).

To capture the target sequences covering whole mitochondrial genome and autosomal SNPs, in solution hybridisation method was used as described by Csáky et al.^35^, Haak et al.^40^ and Lipson et al.^37^. The bait production used for capture was performed based on Fu et al.^41^ and N. Rohland personal communication, with the exemption that the oligos as a pool was ordered from CustomArray Inc. The raw libraries for shotgun sequencing and the captured samples were indexed using universal iP5 and unique iP7 indexes^42^.

Next generation sequencing was performed on Illumina MiSeq System (Illumina) using V3 (2 × 75 cycles) sequencing kits and custom sequencing setup.

### Pre-processing of the Illumina sequence data

Customized in-house analytic pipeline was run on the Illumina sequence data. Paired reads were merged together with SeqPrep master (John JS. SeqPrep. https://github.com/jstjohn/SeqPrep), requiring an overlap at least 10 base pairs for capture, and 5 base pairs for shotgun data. For one mismatch, the one with higher base quality was accepted, the overlapping reads with two or more mismatches were discarded. Cutadapt^43^ were used to remove barcodes as well as to discard fragments too short (< 15 bp for shotgun and < 20 bp for capture) or/and without barcode. The pre-processed reads were mapped to the reference sequence (GRCh37) using BWA v.0.7.5^44^, with MAPQ of 20, and gap extension of 3 base pairs. These permissive options were considered due to the frequent occurrence of low quality and/or amount of reliable fragments in the data pool. Samtools v.1.3.1^45^ were utilized for further data processing, such as indexing or removing PCR duplications. BAM files uploaded to the ENA repository contain both single and paired end reads. Damage pattern estimations were performed by MapDamage v.2.0.6 (https://ginolhac.github.io/mapDamage/). BAM files imported into Geneious 8.1.7 (https://www.geneious.com/) were re-assembled against either rCRS and RSRS using 5 iteration steps. The automatic variant caller of Geneious was used with a minimum variant frequency of 0.8 and minimum coverage of 3 × to collect SNPs to a database. In this step, the known troublesome sites (309.1C(C), 315.1C, AC indels at 515–522, 16182C, 16183C, 16193.1C(C) and 16519) were masked. Remaining ambiguous sites were inspected by eye. Mitochondrial haplogroup determinations were performed in HaploGrep^46^, which utilizes Phylothree mtDNA tree build 17 (https://www.phylotree.org/). The Y-haplogroup were assigned based on Y-STR data using nevgen.org, as well as based on Y-SNP capture and shallow shotgun sequencing data by Y-leaf v1 and v2^47^. Terminal Y-SNPs were verified on the Y tree of ISOGG version 15.34 (https://isogg.org/tree/).

### Estimates of contamination

The contamMix 1.0.10 was used to estimate the level of human DNA contamination in the mitochondrial DNA^40,48^. All of our samples show 99% < endogenous content, which makes them eligible for whole genome analyses. For the results see Supplementary Table S2.

### Population genetic analyses

The different size of populations used in sequence-based analyses is caused by absence of whole mitogenomes of some populations.

Standard statistical methods were used for calculating genetic distances between investigated populations from Ural region (Uyelgi and Cis-Ural) and 26 ancient and 43 modern populations. Nine samples of conquerors from Carpathian Basin were excluded from any population analyses because of the possible sample bias due to selected haplogroups.

The whole mitochondrial genome alignment of the samples were performed in SeaView by ClustalO^49^ with default options, and later regions with poor alignment quality were discarded. Population pairwise F_ST_ values were calculated based on 4015 modern-day and 1132 ancient whole mitochondrial sequences using Arlequin 3.5.2.2^50^ with 10,000 permutations and significance level of 0.05. The Tamura and Nei substitution model was used^51^ with gamma value of 0.62 in case of comparison between two investigated populations from Ural region and 43 modern-day Eurasian populations, For the comparison of 28 ancient populations the F_ST_ calculation was performed with gamma value of 0.599 (for the references see Supplementary Tables S5, S6). The genetic distances of linearized Slatkin F_ST_ values^52^ were used for multidimensional scaling (MDS) and visualized on a two-dimensional plots (Supplementary Figs. S9a and S10) using metaMDS function based on Euclidean distances implemented in the vegan library of R 3.4.1^53^.

Spearman rank correlation matrix of F_ST_ values was calculated in Pandas (Python) and visualized in seaborn package by clustermap function using Euclidean metric.

To assess the correlation between genetic and geographic distances for the Hungarian conquerors and two investigated Uralic populations, we performed Mantel test^54^ based on population pairwise F_ST_ and linearized Slatkin F_ST_ values using Arlequin 3.5.2.2^50^ (Supplementary Table S12).

Principal component analysis was performed based on mtDNA haplogroup frequencies of 64 modern and 50 ancient populations. 32 mitochondrial haplogroups were considered in PCA of ancient populations, while in PCA of modern populations and two ancient populations from Ural region we considered 36 mitochondrial haplogroups (Supplementary Tables S3, S4). The PCAs were carried out using the prcomp function in R 3.4.1 and visualised in a two-dimensional plot with first two (PC1 and PC2) or the first and third principal components (PC1 and PC3) (Fig. 2b, Supplementary Figs. S5, S7).

For hierarchical clustering, Ward type algorithm^55^ and Euclidean measurement was conducted based on haplogroup frequencies of ancient and modern populations as well, and displayed as a dendrogram in R3.4.1 (Supplementary Figs. S6, S8). The same population-pool was used as in PCAs.

Shallow shotgun and captured nuclear DNA sequences were 1 bp trimmed on both ends by trimBam function of bamUtil (https://genome.sph.umich.edu/wiki/BamUtil:_trimBam). Genotypes from shotgun data were called for the Human Origin SNP panel by samtools mpileup command (-q30 and –Q30) and by pileupCaller (which is designed to sample alleles from low coverage sequence data, see https://github.com/stschiff/sequenceTools). Methods of genomic PCA are presented in Supplementary Information, Chapter 3. Prior to the ADMIXTURE analysis, we filtered for missing SNPs in the dataset (“-geno 0.999 parameter”) and pruned SNPs in strong linkage disequilibrium with each other using the parameters “-indep-pairwise 200 25 0.4” in PLINK^56^, leaving 1,146,167 SNPs. We run unsupervised ADMIXTURE with K = 16 on a “1240k” worldwide dataset (https://reich.hms.harvard.edu/datasets) of published ancient captured/shotgun sequenced and modern deep sequenced genomes^57^. The plotted samples’ sources are seen in Supplementary Table S10.

### Kinship analyses

We used READ^58^ and lcMLkin^59^ software on our samples, to assess IBD patterns for nuclear data. The informative positions do not exceed 400 SNPs on pairwise comparisons, which are below of comparable thresholds for each software.

### Phylogenetic and network analysis

All available mitochondrial genome sequences in NCBI (more than 33,500) were downloaded and sorted according to their haplogroup assignments. Then multiple alignments for each haplogroup were performed with ClustalO within SeaView^49^. Neighbour Joining (NJ) trees were generated by PHYLIP version 3.6^60^. The phylogenetic trees then were drawn by Figtree version 1.4.2 (https://tree.bio.ed.ac.uk/software/figtree). We decided to omit median joining network (MJN) to avoid unresolvable ties and bootstrap calculation due to the low number of substitutions.

To analyse the Y-STR variation within the Y-chromosomal haplogroups N1a1-M46 and G2a, Median Joining (MJ) networks were constructed using the Network 5.0 software (https://www.fluxus-engineering.com). For the N1a1-M46 Y-haplogroup MJ network calculation with 17 STR loci 238 samples, and for MJ network calculation with 12 STR loci of the same haplogroup, 335 samples of 27 ancient and modern population were included (Supplementary Table S8). The MJ network analysis of G2a Y-haplogroup was calculated based on 14 STR data using 120 samples of 27 populations (Supplementary Table S9). Post processing MP calculation was used, creating network containing all shortest tree. Repeats of the locus DYS389I were subtracted from the DYS389II.

## Supplementary information

Supplementary Tables.

Supplementary Figures.

## Data Availability

The NGS data were uploaded to the repository ENA (European Nucleotide Archive) under Project No. PRJEB39054.

## References

[1] Ivanov, A. *Drevnie ugri-madyari v Vostochnoi Evrope* (1999).

[2] Botalov, S. G. Pogrebalnii kompleks Uyelgi i nekotorie nabludeniya na predmet ugorskogo i madyarskogo kulturgeneza. in *A népvándorláskor fiatal kutatóinak XXIV. konferenciája Esztergom 2014. november 4–6. II.* (ed. Türk, A.) 267–334 (Studia ad Archaeologiam Pazmaniensiae No. 3.2—Magyar Őstörténeti Témacsoport Kiadványok 3.2., 2017).

[3] Belavin, A. M., Ivanov, V. A. & Krilasova, N. B. *Ugri v Preduralya v drevnosti*. (2009).

[4] Komar, A. Istoriya i arheologiya drevnih madyar v epohu migratsii—A korai magyarság vándorlásának történeti és régészeti emlékei. in *Studia ad Archaeologiam Pazmaniensia 11.—Magyar Őstörténeti Témacsoport Kiadványok 5.* (eds. Türk, A. & Budai, D.) (2018).

[5] Tyurk, A. Vozmozhnosti i perspektivi arheologicheskih issledovanii rannei istorii ugro-madyarov. in *Arheologicheskoe nasledie Urala: ot pervih otkritii k fundamentalnomu nauchnomu znaniyu (XX Uralskoe arheologicheskoe soveshanie). Materiali Vserossiyskoi nauchnoi konferentsii s mezhdunarodnim uchastiem. 25‒29 oktyabrya, 2016 g* (ed. Goldina, R. D.) 268–272 (2016).

[6] Fodor, I. *Vengri: drevnyaya istoriya i obretenie Rodyini* (2015).

[7] Róna-Tas, A. *Hungarians and Europe in the early middle Ages: an introduction to early Hungarian history* (1999).

[8] Hajdú, P. *Uralskie yazik i narodi* (1985).

[9] Klima, L. *Jürkák, tormák, merják: Szemelvények a finnugor nyelvű népek történetének korai forrásaiból.* (MTA BTK magyar Őstörténeti Témacsoport – Források és Tanulmányok 1., 2016).

[10] Kristó, G. *Hungarian History in the ninth Century* (1996).

[11] Türk, A. & Füredi, Á. Latest archaeological results on the origin of the Hungarian people in the Eurasian context. in *IV International Congress of Archeology of the Eurasian Steppes “Nomadic Empires of Eurasia in Archaeological and Interdisciplinary studies”, dedicated to the 100th anniversary of the Russian academic archeology* (eds. Bazarov, B. V & Kradin, N. N.) 93–96 (2019).

[12] Tömöry G (2007). Comparison of maternal lineage and biogeographic analyses of ancient and modern Hungarian populations. Am. J. Phys. Anthropol..

[13] Csősz A (2016). Maternal genetic ancestry and legacy of 10th century AD Hungarians. Sci. Rep..

[14] Neparáczki E (2018). Mitogenomic data indicate admixture components of Central-Inner Asian and Srubnaya origin in the conquering Hungarians. PLoS ONE.

[15] Fóthi, E. *et al.* Genetic analysis of male Hungarian Conquerors: European and Asian paternal lineages of the conquering Hungarian tribes. *Archaeol. Anthropol. Sci.* **12**, 31 (2020).

[16] Neparáczki E (2019). Y-chromosome haplogroups from Hun, Avar and conquering Hungarian period nomadic people of the Carpathian Basin. Sci. Rep..

[17] Post H (2019). Y-chromosomal connection between Hungarians and geographically distant populations of the Ural Mountain region and West Siberia. Sci. Rep..

[18] Bermisheva MA, Tambets K, Villems R, Khusnutdinova EK (2002). Diversity of mitochondrial DNA haplogroups in ethnic populations of the Volga—Ural region. Mol. Biol..

[19] Tambets K (2004). The Western and Eastern Roots of the Saami—the Story of Genetic “Outliers” Told by Mitochondrial DNA and Y Chromosomes. Am. J. Hum. Genet..

[20] Derbeneva OA, Starikovskaya EB, Wallace DC, Sukernik RI (2002). Traces of early Eurasians in the Mansi of Northwest Siberia revealed by mitochondrial DNA analysis. Am. J. Hum. Genet..

[21] Tambets K (2018). Genes reveal traces of common recent demographic history for most of the Uralic-speaking populations. Genome Biol..

[22] Malyarchuk B (2018). Whole mitochondrial genome diversity in two Hungarian populations. Mol. Genet. Genom..

[23] Botalov SG (2012). Novie aspekti i perspektivi v issledovanii problemi «Magna Hungaria». Vestn. Chelyabinskogo gosudorstvennogo Univ..

[24] Goldina, R. D. *Nevolinskii mogilnik VII‒IX vv. v Permskom Preduralye*. (2012).

[25] de Barros Damgaard P (2018). 137 ancient human genomes from across the Eurasian steppes. Nature.

[26] de Barros Damgaard P (2018). The first horse herders and the impact of early Bronze Age steppe expansions into Asia. Science (80–).

[27] Lamnidis TC (2018). Ancient Fennoscandian genomes reveal origin and spread of Siberian ancestry in Europe. Nat. Commun..

[28] Jeong C (2019). The genetic history of admixture across inner Eurasia. Nat. Ecol. Evol..

[29] Grudochko, I. V., Botalov, S. G., Gazizova, S. R. & Tyurk, A. Khronologiya mogilnika Uyelgi (sravnie radiouglerodnih i arheologicheskih datirovok). in *Drevnie i srednevekovie obshestva Evrazii: perekryostok kultur, posvyashennii pamyati vidnogo uchonogo-arheologa, professora, akademika Akademii nauk Respubliki Bashkortostan, doktora istoricheskih nauk Niyaza Abdulhakovicha Mazhitova* (ed. Urazova, A. I.) 78–84 (2018).

[30] Grudochko, I. V. & Botalov, S. G. Novie materiali po kulturogenezu srednevekovogo naseleniya Yuzhnogo Urala (po materialam mogilnikov Uyelgi i Sineglazovo). in *Madyari v Severomu Podniprovji.* 79–99 (2011).

[31] Langó, P. & Siklósi, Z. 10. századi temető Balatonújlak-Erdő-dűlőn (Ein Gräberfeld des 10. Jahrhundert in Balatonújlak-Erdő-dűlő). in *A honfoglalás kor kutatásának legújabb eredményei. Tanulmányok Kovács László 70. születésnapjára. (Monográfiák a Szegedi Tudományegyetem Régészeti Tanszékéről 3.)* (eds. Révész, L. & Wolf, M.) 143–160 (2013).

[32] Balogh, C. Kora Árpád-kori szállási temető Makó-Igási-Járandóban (Campsite Burial Ground from the Early Árpádian Age at Makó-Igási-Járandó). in *Népek és kultúrák a Kárpát-medencében.* (ed. Bollók, Á.) 391–421 (2016).

[33] Ilumäe A-M (2016). Human Y chromosome haplogroup N: a non-trivial time-resolved phylogeography that cuts across language families. Am. J. Hum. Genet..

[34] Fehér T (2015). Y-SNP L1034: limited genetic link between Mansi and Hungarian-speaking populations. Mol. Genet. Genomics.

[35] Csáky V (2020). Genetic insights into the social organisation of the Avar period elite in the 7th century AD Carpathian Basin. Sci. Rep..

[36] Finocchio A (2018). A finely resolved phylogeny of Y chromosome Hg J illuminates the processes of Phoenician and Greek colonizations in the Mediterranean. Sci. Rep..

[37] Lipson M (2017). Parallel paleogenomic transects reveal complex genetic history of early European farmers. Nature.

[38] Dabney J (2013). Complete mitochondrial genome sequence of a Middle Pleistocene cave bear reconstructed from ultrashort DNA fragments. Proc. Natl. Acad. Sci. U. S. A..

[39] Rohland N, Harney E, Mallick S, Nordenfelt S, Reich D (2015). Partial uracil-DNA-glycosylase treatment for screening of ancient DNA. Philos. Trans. R. Soc. Lond. B. Biol. Sci..

[40] Haak W (2015). Massive migration from the steppe was a source for Indo-European languages in Europe. Nature.

[41] Fu Q (2013). DNA analysis of an early modern human from Tianyuan Cave, China. Proc. Natl. Acad. Sci..

[42] Meyer M, Kircher M (2010). Illumina sequencing library preparation for highly multiplexed target capture and sequencing. Cold Spring Harb. Protoc..

[43] Martin M (2011). Cutadapt removes adapter sequences from high-throughput sequencing reads. EMBnet.journal.

[44] Li H, Durbin R (2010). Fast and accurate long-read alignment with Burrows–Wheeler transform. Bioinformatics.

[45] Li H (2009). The Sequence Alignment/Map format and SAMtools. Bioinformatics.

[46] Weissensteiner H (2016). HaploGrep 2: mitochondrial haplogroup classification in the era of high-throughput sequencing. Nucleic Acids Res..

[47] Ralf A, González DM, Zhong K, Kayser M (2018). Yleaf: software for human Y-chromosomal haplogroup inference from next-generation sequencing data. Mol. Biol. Evol..

[48] Fu Q (2013). A revised timescale for human evolution based on ancient mitochondrial genomes. Curr. Biol..

[49] Gouy M, Guindon S, Gascuel O (2010). SeaView Version 4: a multiplatform graphical user interface for sequence alignment and phylogenetic tree building. Mol. Biol. Evol..

[50] Excoffier L, Lischer HEL (2010). Arlequin suite ver 3.5: a new series of programs to perform population genetics analyses under Linux and Windows. Mol. Ecol. Resour..

[51] Tamura K, Nei M (1993). Estimation of the number of nucleotide substitutions in the control region of mitochondrial DNA in humans and chimpanzees. Mol. Biol. Evol..

[52] Slatkin M (1995). A measure of population subdivision based on microsatellite allele frequencies. Genetics.

[53] R Core Team. *R: A language and environment for statistical computing* version: 3.4.3 (R Foundation for Statistical Computing. Vienna). https://www.r-project.org/ (2017). Accessed 8 March 2018.

[54] Mantel N (1967). The detection of disease clustering and a generalized regression approach. Cancer Res..

[55] Ward JH (1963). Hierarchical grouping to optimize an objective function. J. Am. Stat. Assoc..

[56] Purcell S (2007). PLINK: a tool set for whole-genome association and population-based linkage analyses. Am. J. Hum. Genet..

[57] Alexander DH, Novembre J, Lange K (2009). Fast model-based estimation of ancestry in unrelated individuals. Genome Res..

[58] Monroy Kuhn JM, Jakobsson M, Günther T (2018). Estimating genetic kin relationships in prehistoric populations. PLoS ONE.

[59] Lipatov, M., Sanjeev, K., Patro, R. & Veeramah, K. R. *Maximum Likelihood Estimation of Biological Relatedness from Low Coverage Sequencing Data*. *bioRxiv* (2015) 10.1101/023374.

[60] Felsenstein J (1989). PHYLIP—Phylogeny Inference Package (Version 3.2). Cladistics.

